# Author Correction: A generic approach to study the kinetics of liquid–liquid phase separation under near-native conditions

**DOI:** 10.1038/s42003-021-01853-4

**Published:** 2021-03-02

**Authors:** Joris Van Lindt, Anna Bratek-Skicki, Phuong N. Nguyen, Donya Pakravan, Luis F. Durán-Armenta, Agnes Tantos, Rita Pancsa, Ludo Van Den Bosch, Dominique Maes, Peter Tompa

**Affiliations:** 1 VIB-VUB Center for Structural Biology, VIB, Brussels, Belgium; 2 Structural Biology Brussels, Vrije Universiteit Brussel, Brussels, Belgium; 3 Department of Biology, College of Natural Sciences, Cantho University, Can Tho, Vietnam; 4 VIB, Center for Brain & Disease Research, Laboratory of Neurobiology, Leuven, Belgium; 5 KU Leuven, Department of Neurosciences, Experimental Neurology and Leuven Brain Institute (LBI), Leuven, Belgium; 6 Institute of Enzymology, Research Centre for Natural Sciences, Budapest, Hungary

**Keywords:** Biophysical methods, Organelles

Correction to: *Communications Biology* 10.1038/s42003-020-01596-8, published online 19 January 2021.

In the original published version of the Article, in the “Introduction” section, (page 2, first column, fourth line from bottom), the full form of MBP is given as “myelin basic protein”. The correct full form of MBP however should be “maltose binding protein”. The error has been corrected in the PDF and HTML versions of the Article.

