# Supplementary material for: A generic approach to study the kinetics of liquid–liquid phase separation under near-native conditions
Source: Commun Biol. 2021 Jan 19;4:77. doi: 10.1038/s42003-020-01596-8 (PMC7815728; doi:10.1038/s42003-020-01596-8)
Supplement: Supplementary file 2 — Supplementary Information [file 42003_2020_1596_MOESM2_ESM.pdf]

A generic approach to study the kinetics of liquid-liquid phase separation under near-native conditions

Supplementary Figures and Tables

Tabe S1 *Amino acid composition of protein constructs used for LLPS studies*

Segments of the four proteins used in the phase-separation study are outlined in Figure S6. Here, the amino acid composition of the segment, and its % content of cationic (basic, Arg + Lys) plus aromatic (Trp, Phe + Tyr) residues, and Lys plus Tyr, are given.

Amino acid	hnRNPA2 LCD		TDP-43 LCD		NUP98 LCD		ERD 14 FL	
	no.	%	no.	%	no.	%	no.	%
Ala (A)	0	0.0	14	9.5	37	7.4	9	4.9
Arg (R)	9	5.8	5	3.4	2	0.4	3	1.6
Asn (N)	16	10.4	20	13.4	37	7.4	1	0.5
Asp (D)	5	3.2	1	0.7	3	0.6	7	3.8
Cys (C)	0	0.0	0	0.0	1	0.2	0	0.0
Gln (Q)	3	1.9	12	8.1	25	5.0	2	1.1
Glu (E)	0	0.0	2	1.4	5	1.0	39	21.1
Gly (G)	71	46.1	38	25.7	97	19.5	9	4.9
His (H)	0	0.0	0	0.0	1	0.2	6	3.2
Ile (E)	0	0.0	2	1.4	6	1.2	5	2.7
Leu (L)	0	0.0	4	2.7	28	5.6	9	4.9
Lys (K)	1	0.6	1	0.7	15	3.0	34	18.4
Met (M)	2	1.3	10	6.8	4	0.8	2	1.1
Phe (F)	8	5.2	8	5.4	50	10	4	2.2
Pro (P)	9	5.8	4	2.7	25	5	16	8.6
Ser (S)	13	8.4	23	15.5	58	11.6	15	8.1
Thr (T)	0	0.0	0	0.0	95	19.1	8	4.3
Trp (W)	0	0.0	3	2.0	0	0.0	0	0.0
Tyr (Y)	17	11.0	1	0.7	3	0.6	1	0.5
Val (V)	0	0.0	0	0.0	6	1.2	15	8.1
**basic + aromatic**	**35**	**22.7**	**18**	**12.1**	**70**	**14.1**	**42**	**22.7**
**Lys + Tyr**	**18**	**11.7**	**2**	**1.4**	**18**	**3.6**	**35**	**18.9**
**Total**	**154**	**100**	**148**	**100**	**497**	**100**	**185**	**100**

Figure S1 *Reversibility of LLPS of hnRNPA2 LCD*

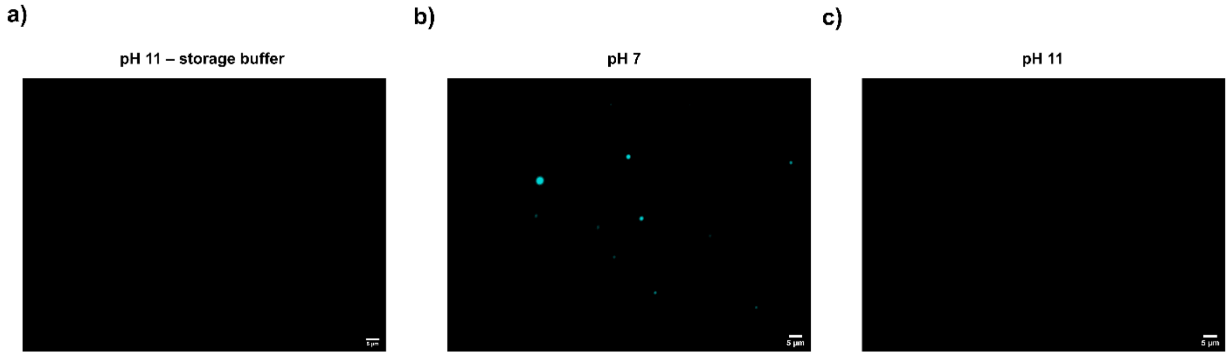

Fluorescence microscopic images of hnRNPA2 LCD were first recorded in its storage buffer (10 mM CAPS, pH 11.0, panel a). Upon decreasing the pH to 7.5, droplets were observed (b). After increasing the pH to 11.0 again, the droplets dissolved (c), showing that LLPS observed with the pH jump method is completely reversible.

Figure S2 *Turbidity changes of hnRNPA2 LCD at different wavelengths*

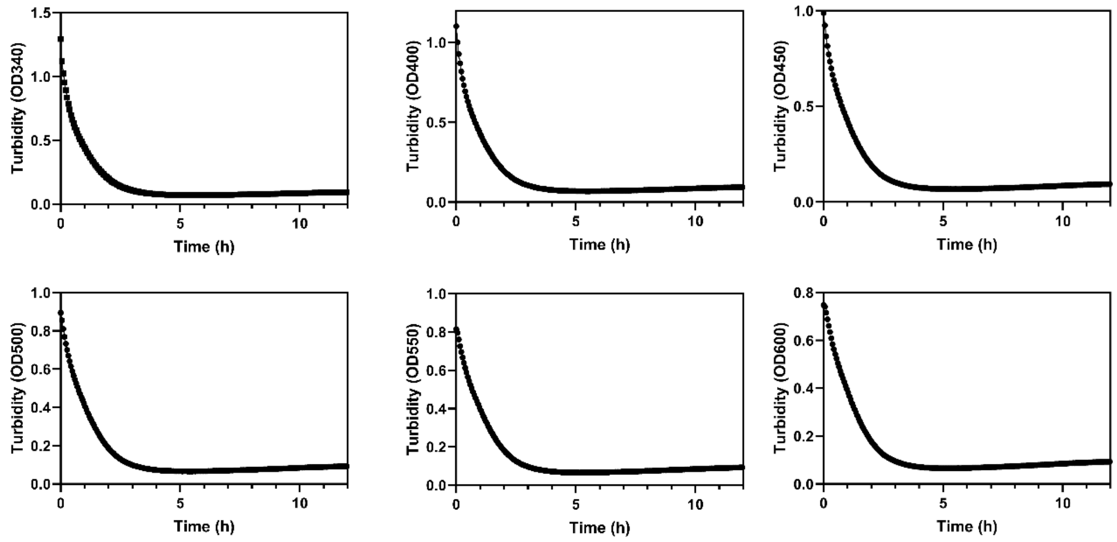

Turbidity measurements after initiating the LLPS of hnRNPA2 LCD by pH jump over a range of wavelengths (340nm, 400nm, 450nm, 500nm, 550nm, 600nm) all show a similar behavior.

Figure S3 *Kinetic artifacts of the LLPS of hnRNPA2 LCD induced by urea dilution and TEV cleavage*

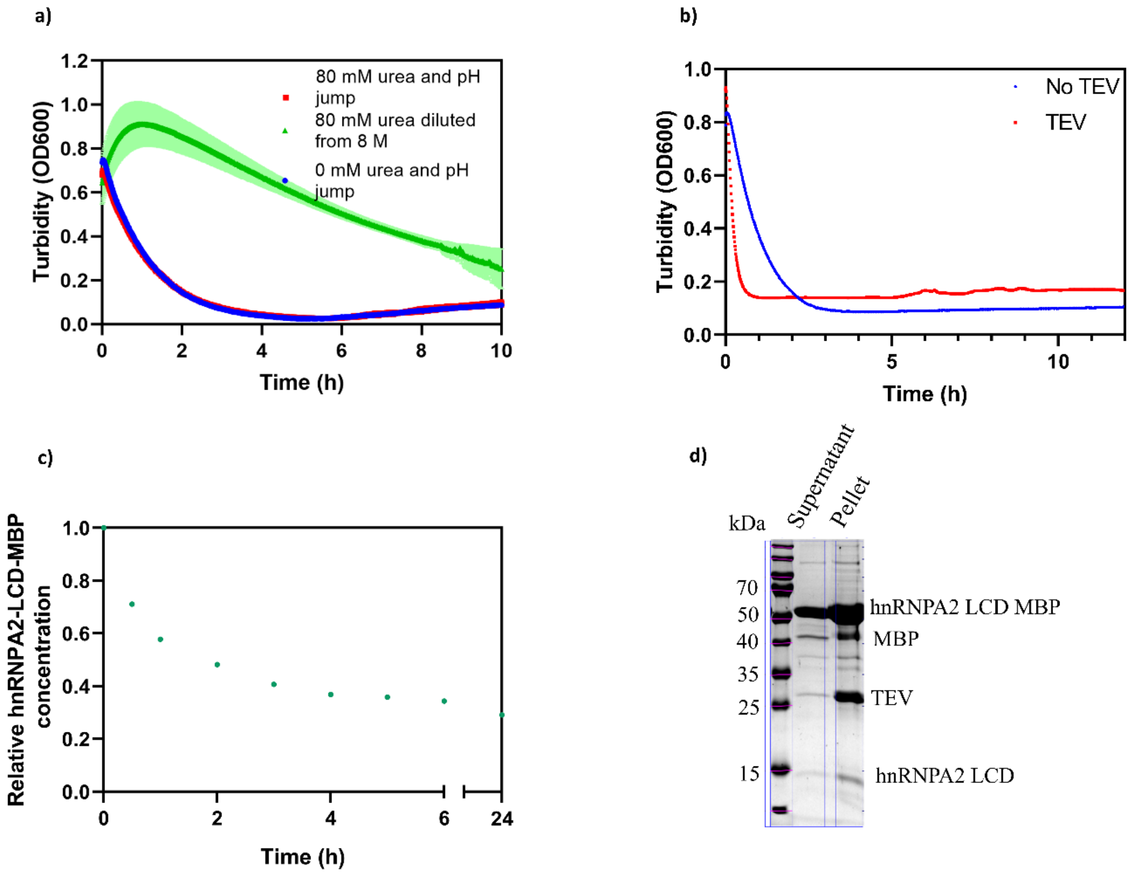

(a) Kinetics of hnRNPA2 LCD LLPS (at 20 μM) was followed by turbidity (OD600) measurements, upon initiating LLPS by a pH jump from 11.0 to 7.5, in the absence and presence of urea. The kinetics strongly differ if a final concentration of 80 mM urea is reached by: (i) 100x dilution from a protein solution stored in 8M urea (green) and (ii) adding 80 mM urea to the protein solution stored at pH 11.0, followed by the pH jump to pH = 7.5 by a small amount of concentrated buffer (red). This latter curve is very similar to the one observed by the pH jump in the absence of added urea (blue). (b) Kinetics of LLPS in the pH jump system was monitored by turbidity measurements in the presence (blue) and absence of TEV (0.2 μM, red). (c) Cleavage of 20 μM hnRNPA2 LCD-MBP by TEV protease (at 1:100 (w/w) ratio) was monitored over time by determining the relative intensity of uncleaved protein by SDS-PAGE and densitometry (for the corresponding LLPS trajectory, cf. Figure 1c, 1f and 1i). Apparently, cleavage comes to a halt when about 40% of the original fusion construct is still uncleaved. (d) The supernatant and pellet of the sample were separated about 20 min after starting the reaction and run on SDS PAGE, showing that most of cleaved MBP and the enzyme TEV gets trapped in droplets. Please note that hnRNPA2 LCD stains bad by Coomassie, but is missing from the supernatant.

Figure S4 *Stopped-flow kinetics of the LLPS of hnRNPA2 LCD*

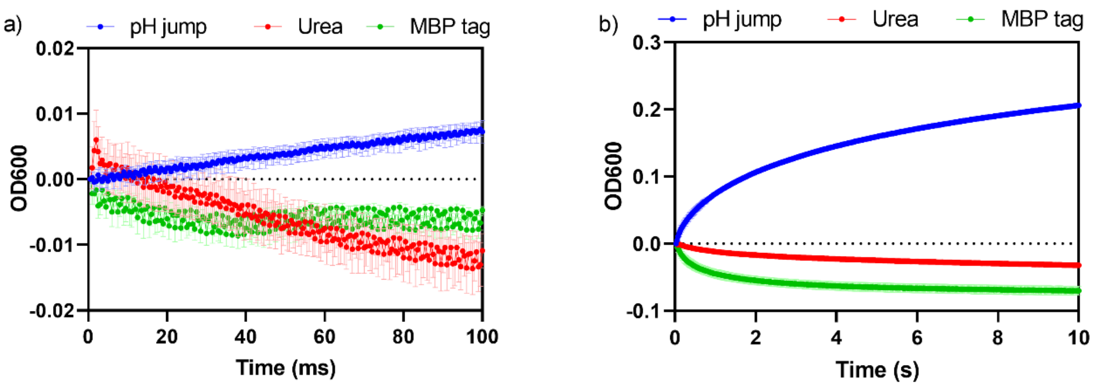

LLPS of hnRNPA2 LCD (at 20 μM) was initiated by the three methods, pH jump (blue), urea dilution (red) and TEV-cleavage of MBP tag (green), as described in Methods. The change of absorbance at 600 nm (OD600) was monitored by stopped flow for 100 ms (a) and 10 s (b). Three measurement with 2 recordings each have been averaged, mean ± SD are shown.

Figure S5 *Selection of an appropriate solution pH for studying LLPS by a pH jump*

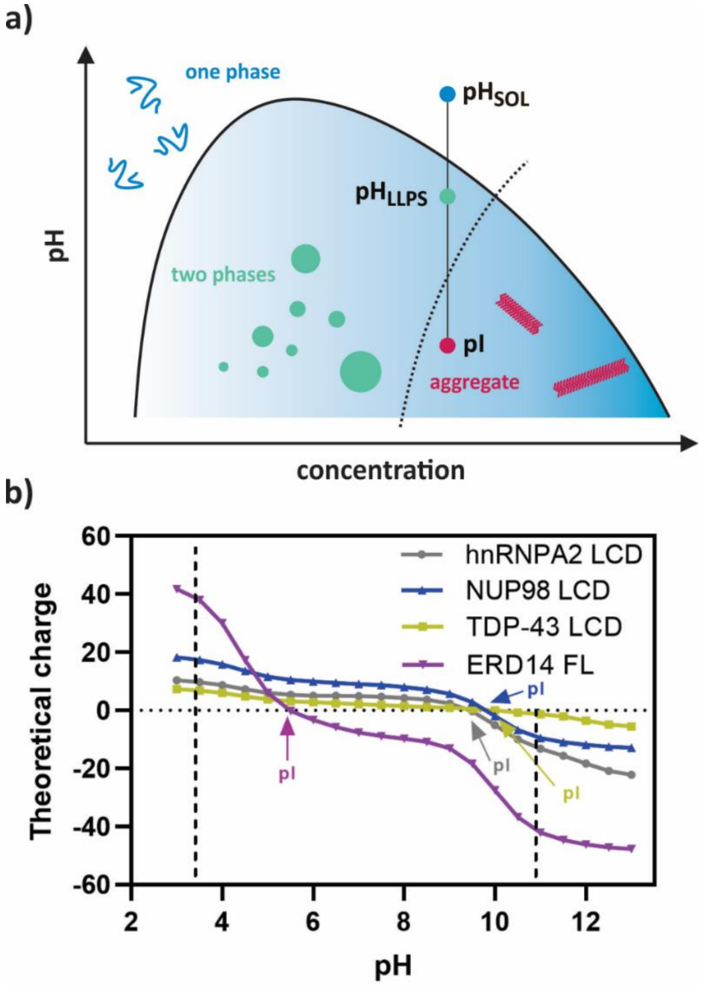

General phase diagram of proteins in the pH vs. concentration plane, illustrating the LLPS response of an aggregation-prone protein and the principle of selecting an appropriate pH value for keeping it in solution and initiating its LLPS by a pH jump. pH has been noted as a primary regulator of LLPS and subsequent aggregation at deep supersaturation, i.e. at a pH corresponding to their pI, where their net charge = 0 {Berry, 2018 #42;Shin, 2017 #41;Munder, 2016 #44}. The phase diagram suggests that the protein can be kept in solution at a pH basically different from their pI (pH_{SOL}, in either direction on the pH scale, cf. panel b and also ref. {Adame-Arana, 2020 #46}), when they have a large repulsive net charge. By jumping with their pH to an intermediate value (pH_{LLPS}), they start to phase separate but not to aggregate. (b) The theoretical net charge of the four proteins studied, hnRNPA2 LCD, NUP98 LCD, TDP-43 LCD and full-length ERD14 (cf. Figure S6), was calculated as a function of pH by the http://protcalc.sourceforge.net server. The selected pH_{SOL} values are indicated by vertical dashed lines, and pI values of the proteins (where their net charge = 0) are shown by arrows.

Figure S6 *Domain organization of hnRNPA2, TDP-43, NUP98 and ERD14*

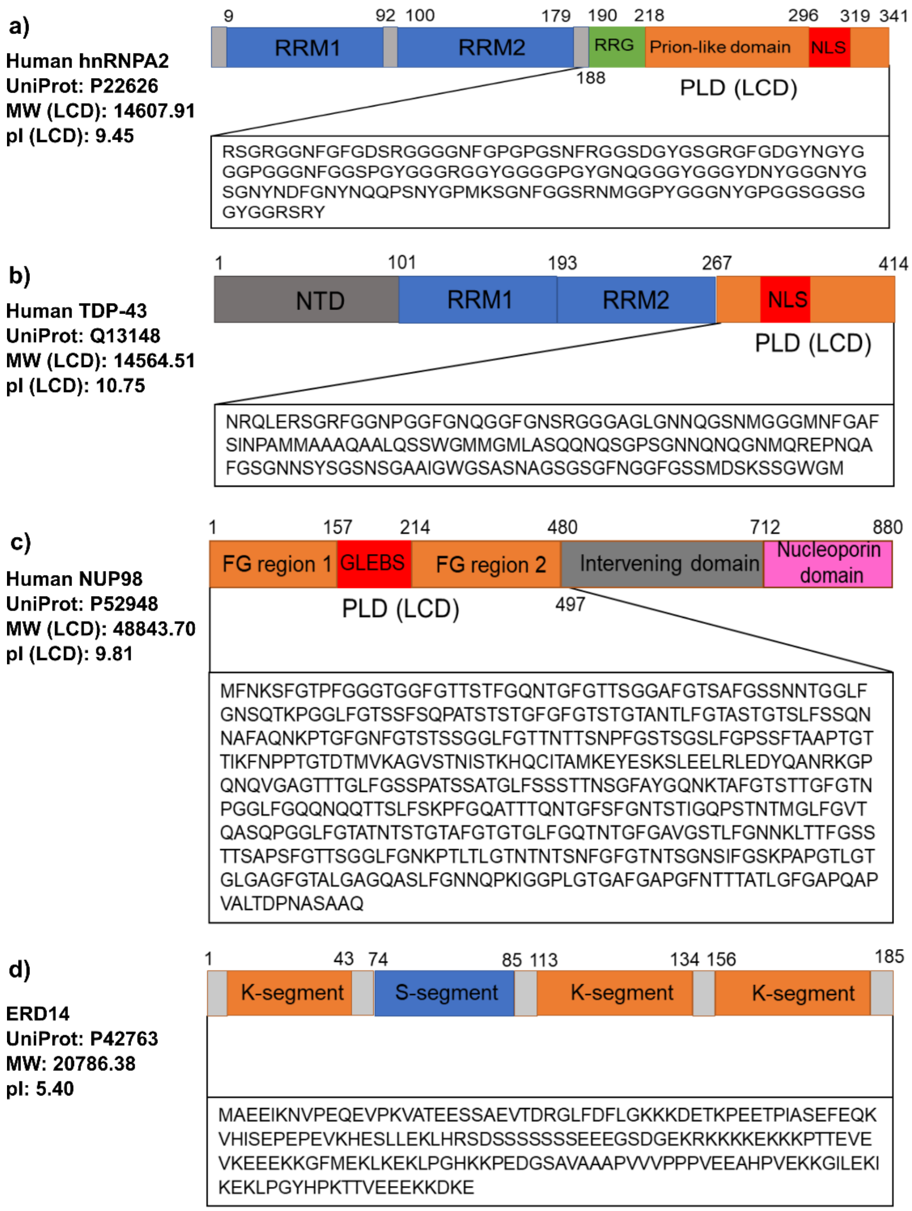

The domain organization of the proteins is taken from UniProt (http://www.uniprot.org/). UniProt ID and UniProt rational name are given. The amino acid sequence and position within the full sequence of the phase-separating region is shown in a blow-up window, along with the Mw and theoretical pI of the segment. Domain abbreviations are: GLEBS, region that binds to RAE1 (mRNA export factor); K-segment, Lys-rich domain; LCD, low-complexity domain; NLS, nuclear localization signal; NTD, N-terminal domain; RRG, Arg-Arg-Gly region; RRM, RNA recognition motif; PLD, prion-like domain; S-segment, Ser-rich domain.

Figure S7 *ERD14 does not phase separate in the absence of poly(U)*

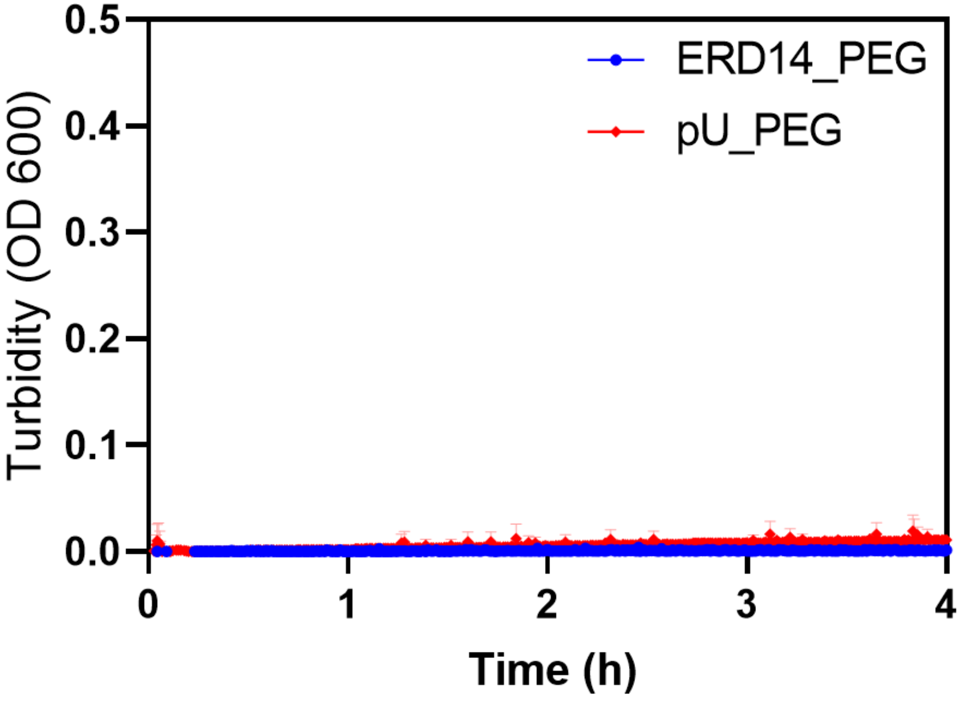

LLPS was monitored by measuring its turbidity (OD600) under the same conditions as on Figure 2f. ERD14 does not undergo LLPS in the presence of PEG but absence of poly(U) (ERD14_PEG, blue line). Poly(U) in the absence of ERD14 also does not phase separate (pU_PEG, red line).

Figure S8 *Raw data of LLPS of hnRNPA2 LCD by dynamic light scattering*

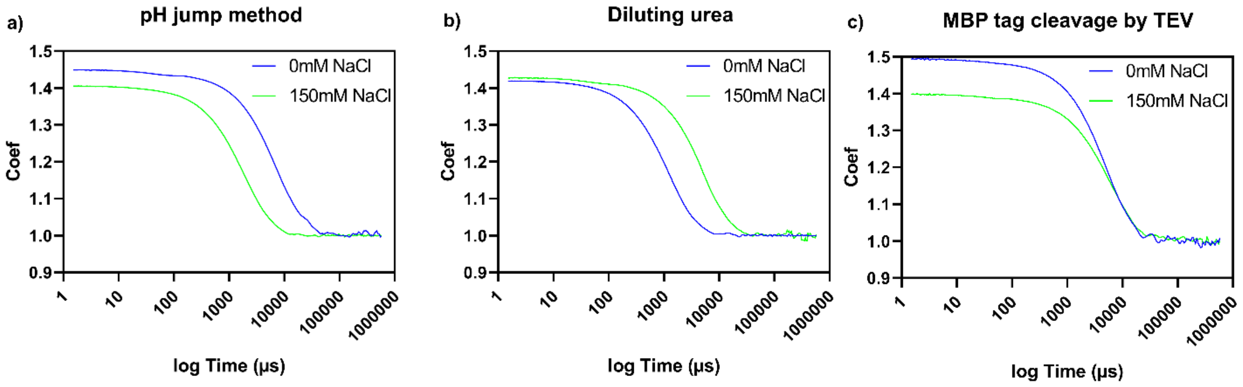

Raw data of the correlation curves obtained by DLS measurements 1h after initiating LLPS of hnRNPA2 LCD by pH jump (a), urea dilution (b) or TEV-cleavage of MBP tag (c). Fits of data were used to create kinetic curves of the change of droplet size, e.g. on Figure 1g, h and i.

Figure S9 *Concentration dependence of liquid-liquid phase separation of the protein constructs used*

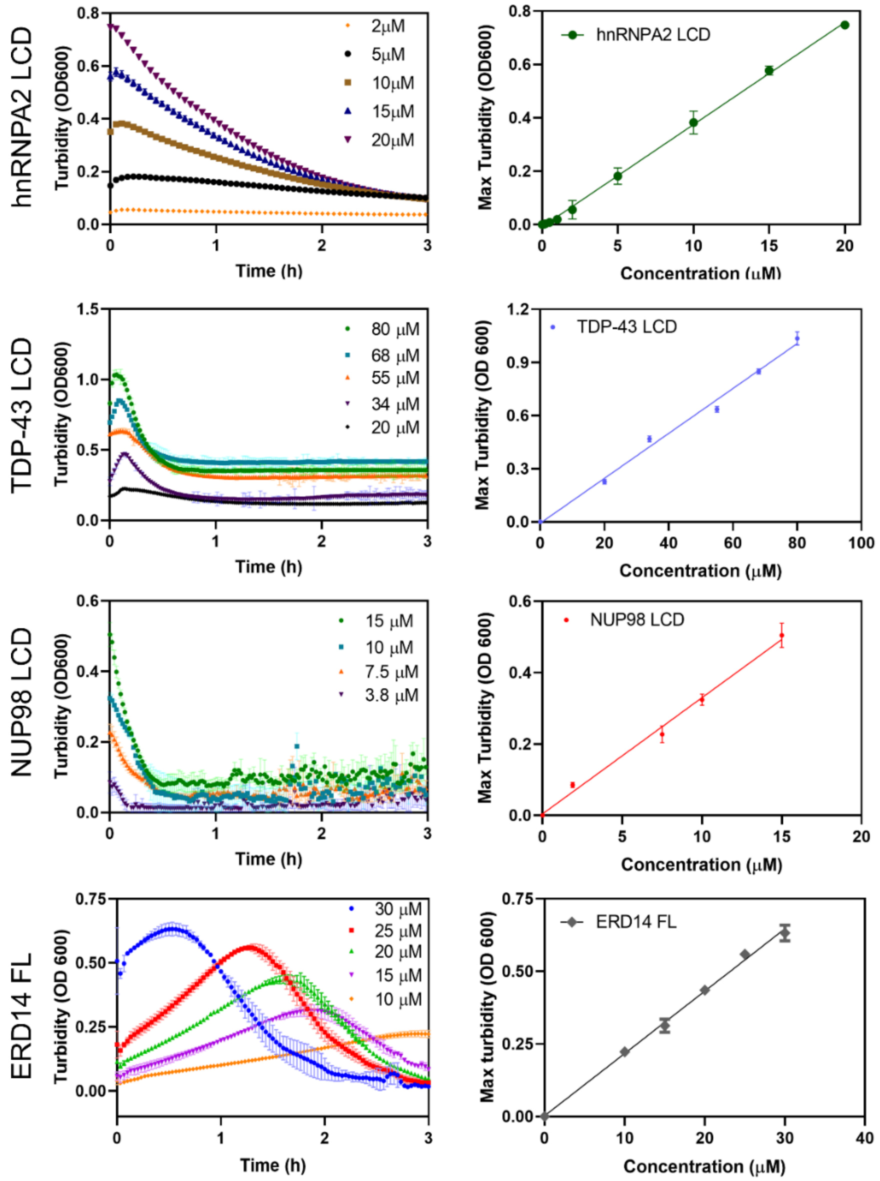

Kinetics of LLPS of the four constructs hnRNPA2 LCD, TDP-43 LCD, NUP98 LCD and full-length ERD14, were monitored by turbidity (OD600) over time at different concentrations (left panels). Peak heights as a function of concentration show a linear dependence on concentration (right panels).

Figure S10 *Characteristic features of the amino-acid composition of protein constructs*

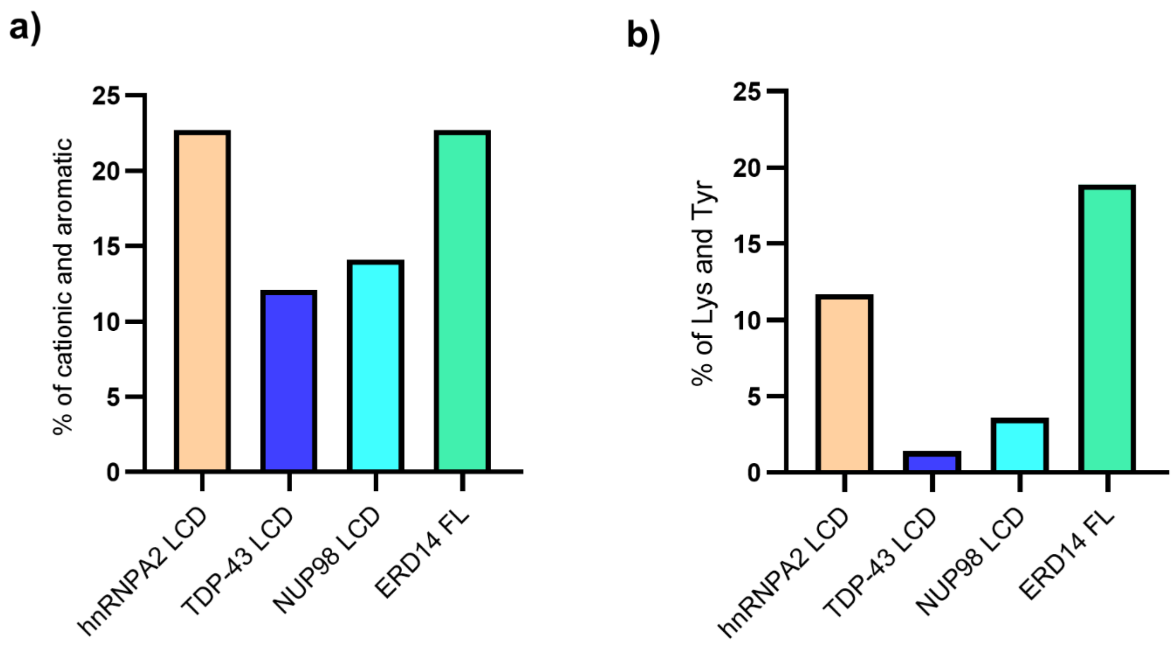

The sequences of protein constructs used in this LLPS study, hnRNPA2 LCD, TDP-43 LCD, NUP98 LCD and ERD14 FL (cf. Figure S6 and Table S1) have been analyzed for the % of cationic (or basic, Arg and Lys) plus aromatic (Trp, Phe and Tyr) residues (a) and the % of Lys plus Tyr (b).

Figure S11 *pH jump of globular control proteins*

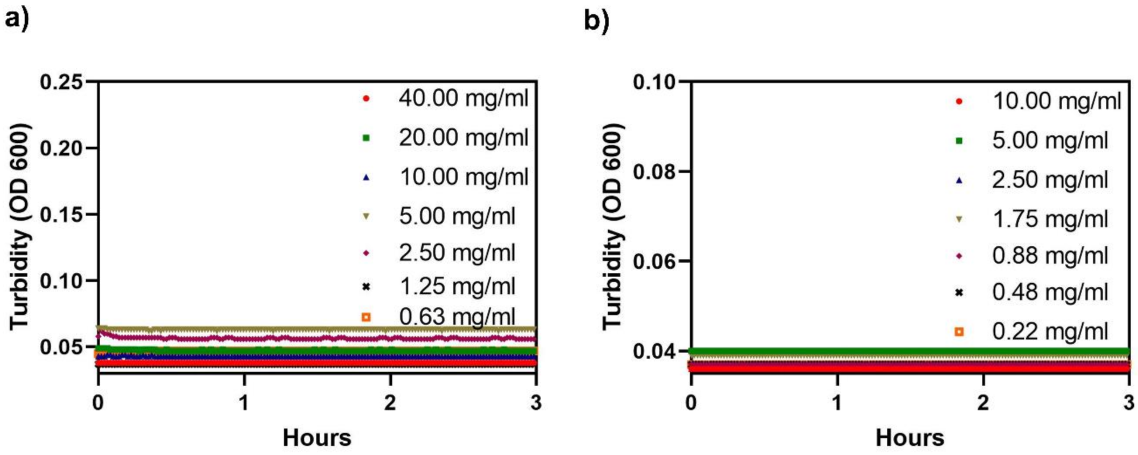

Phase separation of bovine serum albumin (BSA) and lysozyme was assessed by the pH-jump method at physiological pH and a broad range of concentrations. (a) BSA was dissolved in 10 mM CAPS buffer, pH 11.0 at different concentrations, and its pH was changed to 7.4 at t = 0 by the addition a small volume of 0.5 M MES buffer, pH 5.5. (b) Lysozyme was dissolved in 20 mM MES buffer, pH 5.5, and its pH changed to 7.4 at t = 0 by a small volume of 0.5 M CAPS buffer, pH 11.0. Whereas LLPS of both proteins has been repeatedly observed at non-physiological conditions, no sign of LLPS is seen here by measuring turbidity at OD600 at any of the concentrations tested.
