# Supplementary material for: A generic approach to study the kinetics of liquid–liquid phase separation under near-native conditions
Source: Commun Biol. 2021 Jan 19;4:77. doi: 10.1038/s42003-020-01596-8 (PMC7815728; doi:10.1038/s42003-020-01596-8)
Supplement: Supplementary file 3 — Description of Additional Supplementary Files [file 42003_2020_1596_MOESM3_ESM.pdf]

Description of Additional Supplementary Files

File Name: Supplementary Data 1
Description: **Data points of Figure 1**
Data points corresponding to Figure 1, panels Fig. 1d, Fig. 1e, Fig. 1f, Fig. 1g, Fig. 1h and Fig. 1i, are given. For each panel, the curves have been measured at 0 mM NaCl and 150 mM NaCl, and for each datapoint of each measurement, values of “time” “mean” and “standard deviation (SD)” are given.

File Name: Supplementary Data 2
Description: **Data points of Figure 2**
Data points corresponding to Figure 2, panels Fig. 2d, Fig. 2e, Fig. 2f, Fig. 2g, Fig. 2h and Fig. 2i, are given. For each panel, the curves have been measured at 0 mM NaCl and 150 mM NaCl, and for each datapoint of each measurement, values of “time” “mean” and “standard deviation (SD)” are given.
